# Statistics and the Question of Standards

**DOI:** 10.6028/jres.101.074

**Published:** 1996

**Authors:** Stephen M. Stigler

**Affiliations:** Department of Statistics, University of Chicago, Chicago, IL 60637

**Keywords:** least squares, normal distribution, sampling, standards, standards of tolerance, statistics, uncertainty

## Abstract

This is a written version of a memorial lecture given in honor of Churchill Eisenhart at the National Institute of Standards and Technology on May 5, 1995. The relationship and the interplay between statistics and standards over the past centuries are described. Historical examples are presented to illustrate mutual dependency and development in the two fields.

## 1. Introduction

**Fig. 1:**
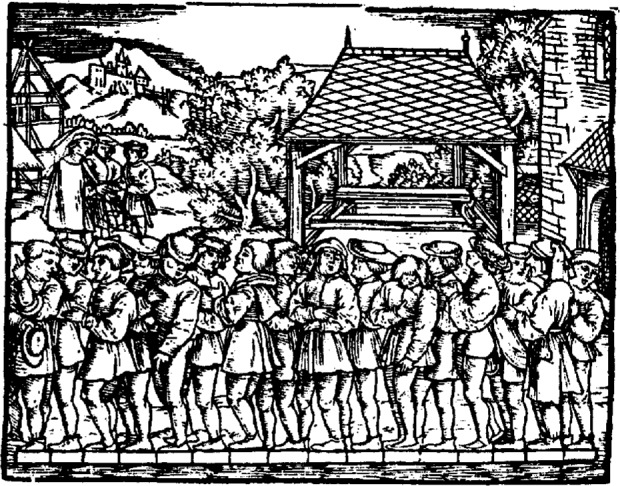
The determination of a “right and lawful rood” or rod in the early sixteenth century in Germany by measuring an essentially random selection of 16 men as they leave church.

Churchill Eisenhart was a good friend for over 20 years—a short span compared to the relationships many of those here at NIST had with him, but the impression he made on me and my work was of crucial importance to me. We met through correspondence in 1972, and the last letter I received from him—less than a month before his death—is at the other end of a 4 inch file from the first. I chose my title with Churchill in mind. When he retired in 1983, I wrote to him that he had set the standard for scholarly research in our field, and that is how I thought of him—the standard. The illustration in Fig. 1 that Mark Levenson^1^ chose to illustrate the announcement for this lecture could not have been more apt. It is a picture from a book published in Frankfort in 1535 or 1536, a book on surveying by Jacob Köbel called, *Geometrei* [1], showing how a “right and lawful rood” or rod of 16 feet should be determined by measuring an essentially random selection of 16 men as they leave church. Churchill owned a personal copy of that book, and he was immensely proud of it.

What is a “Standard”? *The Oxford English Dictionary* (OED) tells us that the word “Standard” in the first sense means “A military or naval ensign,” a usage that is traced back to the year 1138 and the Battle of the Standard. (That is surely a recurring theme around NIST!). The OED tells us that the origin of the other sense of “standard”—as “standard of weight or measure” is, in their words, “somewhat obscure.” I will not shed any light on the evolution of the word “standard” in the sense we employ it today. I expect that both senses long predate the sources even the OED gives, and thus their origins and relations are lost to history. Instead I have a different goal. I will discuss the historical relations of statistical concepts and standards. The discussion will necessarily be highly selective.

I believe the types of numerical standards we discuss today can be roughly described as falling into one of two types, which I might call *goals* and *limits*. The first of these is the standard as basis, as target, as goal, as ideal, as an anchor for comparison. Such are the standards of weight or measure—the meter or the liter, or the so-called physical constants, such as the speed of light. The other type is that of standards as tolerance levels, as limits beyond which one cannot respectably go, such as minimum standards of performance. This type of standard may be so high as to be all but unattainable, or so low as to be ludicrous; the recent discussions of national standards of educational attainment come to mind.

**Fig. 2:**
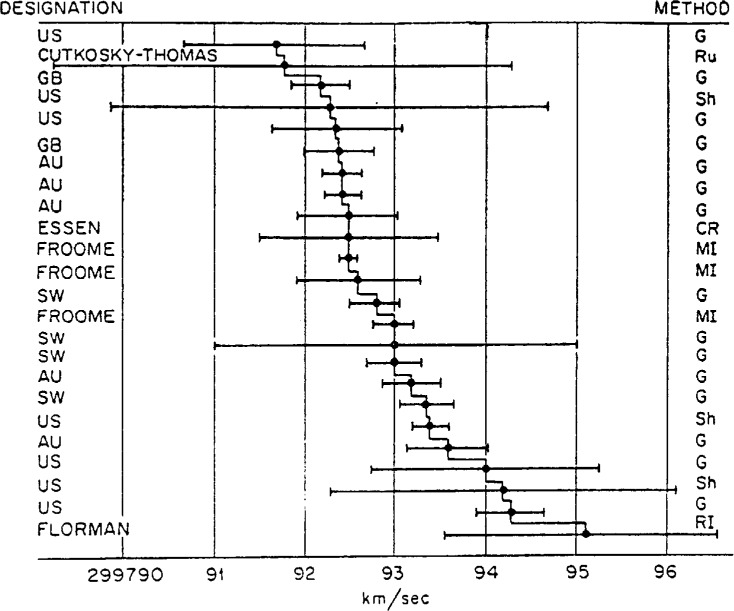
Measurements of the speed of light with the reported errors (from Youden, 1972; giving as the source McNish, 1962).

The two types of standards have features in common. First, their primary use is for comparison. And second, the idea of a standard entails some sense of permanence. We simply would not think of transitory standards as true standards; a standard must be for all time, or at least for some considerable length of time. Yet as this audience knows full well, permanent does not mean never changing. Even the great constants of nature have been known to slip, to shift in ways that contradict common preconceptions. Jack Youden’s famous 1968 address on “Enduring Values” [2] made the point for interlaboratory testing brilliantly by showing how the most fundamental of “constants”—the astronomical unit and the speed of light—have appeared to change over time, as changes of experimental technique have made a mockery of the statisticians’ nominal error bars. Of the 15 measurements of the Astronomical Unit that he presented (Table 1), not a single one fell within the range of possible values given by its immediate predecessor. The variations in the speed of light were not much better (Fig. 2).

A different sort of slippage is at least as common, and more generally known, in regard to standards as limits. Standards of what we will accept (or at least tolerate) evolve for many reasons. Here is an example from military history; the source for these data by the way is Karl Marx’s, *Das Kapital*! [3] (Table 2). So “standards” are not truly “constants.”

My thesis is a very simple one: historically considered, standards and statistics are nearly inseparable. Without the problems of standards in the OED’s second sense (that is, not standards as flags), we would not have modern statistics. And without statistical concepts we would not have standards, much less the National Institute of Standards and Technology. Now, this thesis is so simple as to be almost self-evident, and is surely so congenial to this audience, that I shall not spend long in demonstrating it. I shall give a short and selective historical account, a few examples, and then try to see what counter-evidence might be offered.

### Coinage and Sampling

The history of the relationship of statistics and standards actually goes back as far as either of the two terms have been traced, back at least to the middle of the twelfth century. The first example I have in mind makes the point extremely well. In the century after the Norman invasion of 1066, a monetary system evolved in England where an independent mint, the London Mint, would mint gold coins from ingots furnished by the king, the barons, or by others in trade. Obviously in such a situation there was a need to provide checks on the amount and fineness of the gold in the coinage. The power of the English king was insufficient to merely assert the value of the coins.

A system was set up as early as the year 1150 that was called the Trial of the Pyx, where the mint’s coinage would be put to test [4]. Think for a moment what would be needed for such a test: a standard and statistical methods. A standard would be needed for comparison, for how else to tell if a newly minted coin was as promised? As a standard of fineness, a bar of gold was retained in a safe place as a reference. And statistical methods were needed, for two reasons. First, the sheer volume of the coinage would make individual weighing of each coin extremely difficult, and second, tests of fineness were destructive, making tests of each coin impossible.

So sampling was needed, and being needed, it was invented. The earliest documents are not specific about how the samples would be drawn, but it is impossible to believe that the different (and very suspicious) parties would have been satisfied with a selection that they did not see as essentially random. One description from the year 1280 refers to the coins being placed on a table, then being “well turned over and thoroughly mixed by the hands of the Master of the Mint and the Changer, let the Changer take a handful in the middle of the heap, moving round nine or ten times in one direction or the other, until he has taken six pounds.”

But sampling was not the only statistical method born of necessity in this trial; there were two others of note. One of these was one whose history Churchill specialized in, the Mean. In order to avoid having vaguely understood uncertainties of weighing mask major variations in the weight of coins, the coins were weighed in aggregate, say 100 at a time. Essentially, then, it was the average weight of the tested coins that was compared with the standard. Of course from the point of view of experimental design, this was admirable—the aggregate was subject to one measurement error rather than one hundred. And there was yet another statistical method employed, an allowance for variability. Because mint technology was not perfected, it was granted by all that some allowance had to be made for variability. If the coins weighed too little, the barons were being cheated and the acceptance of the coins in circulation was jeopardized. If the coins weighed too much, the larger coins would be culled from circulation, melted, and recoined, with the profit going to the merchant. Neither situation was tolerable. The allowance that the contract specified was called the Remedy, because measures outside these limits would need to be remedied by the Master of the Mint. In some early documents it stated that the Master was at risk in life and limb!

Let me emphasize the point of this example: Two standards were needed, one of each type: the standard of weight and the standard of tolerance (the Remedy). These standards could not be useful without the statistical methods that were invented for that purpose, and the statistical methods would not have been invented but for the need for the standards. But lest you think that the appearance of all statistical methods is historically inevitable when they are needed, let me tell you one thing more about the Trial of the Pyx, about the method that was not invented, and not invented because of a lack of statistical theory. It is true that at an early time the barons of the exchequer had the notions of sampling, of aggregation, and of allowance for variability. But they did not know how to put them together. Essentially, they specified the allowance for a single coin, then extrapolated it by multiplication to get an allowance for the aggregate. In modern parlance, they multiplied by *n*, not by square root of *n*. They did not know the $\sqrt{n}$ rule, which was only known after De Moivre found it to hold for the binomial in 1731. This left the way open for exploitation, and a limited amount of evidence suggests that there was, indeed, gradual exploitation, as Masters of the Mint discovered how they could slightly short-weight coins and still remain safe. The English testified that the French did this, and I suspect the French felt the same about the English.

### Least Squares and Geodesy

Let me move to another, much more recent example, to one of Churchill’s favorite topics—least squares [5,6]. The story of the discovery of least squares is well-known, but it may not be as widely appreciated as it should be, that the discovery was *specifically* made in pursuit of the solution of a problem in standards. In the 1790s, in the aftermath of the French Revolution, the French decided to create a new system of weights and measures, the metric system. The base, the standard of the metric system, was to be the meter, and the definition of the meter was to be such that the length of a meridian quadrant, the distance from the North Pole to the Equator along the surface of the Earth, was to be 10 000 000 meters. And on “purely objective” grounds, the French determined that not just *any* meridian quadrant would do—it was to be the meridian quadrant that passed through the Observatory of Paris. Teams were dispatched to measure the arc from Paris to Barcelona, and it fell to the mathematicians in Paris to complete the calculation, to reduce the observations, to determine the ellipticity of this arc, and to find once and for all time the standard meter. The mathematician most closely involved was Adrian Marie Legendre. And in the course of this study, Legendre discovered least squares. Most historical accounts emphasize the role of astronomy in the development of least squares, but it is clear that, despite the fact that Legendre published his discovery in a book on the orbits of comets, it was the determination of the meter that had inspired him. Even in the 1805 book on comets, the only worked example of least squares—and the only published example for several years afterwards—was Legendre’s analysis of the data on the French meridian arc.

So least squares owes its discovery to standards. It would perhaps be too much to claim that if the French had not needed the length of the meter, least squares would not have appeared about that time. But I think the converse is valid: The need for the meter called forth the method of least squares, just as the measurement of a different meridian arc had brought forward the method of least deviations regression a half-century earlier in the work of Boscovich [6,7]. The French had seen a need for a precise determination, a need that could not be met by available methods, and the best minds of the time had responded with the single most useful statistical method of all time. A problem of standards had called forth statistical creativity, and through statistics the standard meter was created. But it is worth pausing here to note what did not appear. In 1805 Legendre had given powerful expression to the method of least squares, but not to an assessment of its accuracy.

In fact, there was *no* probability in Legendre’s least squares. Only with the work of Gauss, not published until 4 years later, do we find probability: the normal distribution, standard errors of coefficients, weighted analyses. What could account for this strange discrepancy? Was it only, as some historical accounts might seems to suggest, only a reflection of the fact (which few would dispute) that Gauss was a better mathematician than Legendre? I venture to suggest a different explanation. It is simply this: Legendre’s basic charge was to determine a *single* standard, the meter, through the most precise analysis of the observational data relevant to the problem. He sought the most precise French meter, based on French data on the French arc. He did not use other available data from outside France, even though they would have afforded a more accurate reading on the ellipticity and therefore on the meter. Indeed, there is a sense in which an assessment of uncertainty would have been detrimental to his mission. Can you imagine how happy Napoleon would have been if Legendre had come in with two bars in hand and announced, “Sire! We have found the meter—it is somewhere in length between this bar and this bar!”

Gauss on the other hand was primarily interested in problems in astronomy where uncertainty was a staple of life. My argument has the following corollary: problems in the determination of basic standards (the first of my two types) may have inspired the heights of statistical ingenuity, but the precise determination of basic physical constants and the determination of the uncertainty in those determinations are uneasy bedfellows. Admission of the second of these (uncertainty) can be seen by the insecure as undermining the first. That of course was a message that Churchill spent much energy on, trying to encourage researchers to feel more secure in facing an uncertain world [8], and it was one of Youden’s messages as well [2]. I have seen abundant historical evidence to support this corollary. Some time ago I was engaged in an historical retrospective on the performance of modern estimators on ancient data sets. I readily found examples from astronomers, but when I looked for some in chemists’ early attempts to determine molecular weights, I was surprised by what I found. Without exception, the best work I found gave great detail on the experiments, but it only reported numerical results for the handful of experimental runs that gave the favored answer, except for the last one or two decimal places.

I have mentioned some areas in statistics where statistical innovation has been inspired by problems of standards, and one (the assessment of uncertainty of constants) where the historical relationship is not of this sort. Are there other examples where statistics and standards have not gone hand in hand? Yes, I would submit that the entire area of the measurement of statistical association grew up quite distant from the study of standards, and surely there are many others. This is not to say that there are any areas in statistics that are not happily at home in the modern NIST, only that there are some that did not grow to maturity either at NIST or in one of its cousin institutes around the world.

I want to comment on two other aspects of the relationship of statistics and standards, and then present a curious historical example of a statistical construct that has itself become a standard. The first of these two aspects is the way statistical considerations have determined the very notion of what is a “standard”; the second is the way the lack of a commonly understood “standard of statistics” has impeded the development of the subject of statistics. The first of these is of course well known (in at least some instances) to this audience. In 1805, the meter was a simple length, based upon the magnitude of the Earth as determined by the best scientists of France and thus of the world. In 1890 the meter was a metal bar resting comfortably in Paris, and the speed of light was a time determined from distance and angular measurements that were in turn derived from that metal bar. The bar was the standard of distance and indirectly of all else. In 1984 the meter was no longer the primary standard; light itself had taken over that role. In 180 years there had been a revolution of standards, from Earth to bar to light. What had happened?

Here is another example, commonplace in some respects, exotic in others. Currently, a price index is simply this: A commodity or package of commodities is defined, and the price to obtain that commodity is determined. That price is then followed over time. The commodity (or package) is the standard; the price is transient, changing as the world changes, perhaps daily. But it was not always so. Look at this example from more than 3 centuries ago (Figs. 3a and 3b). In 1665 London suffered through the last of the great plagues. A statistical offshoot of the plagues was the weekly publication of the Bills of Mortality, primarily lists of deaths, classified by region and by cause. But the Bills of Mortality also included something for the living—birth statistics and a price index. The price index was subtly different from what we have now. It gave the weekly amount of bread a penny would buy. In 1665 the penny was the standard, and the amount of bread was transient, the reverse of what we have now, the same kind of reversal that took place in the past century between the meter and the speed of light. I submit that statistical considerations underlie both reversals.

The principle is very simple. Standards are for comparison, and the surest, best-anchored, best-determined quantity serves as the best standard. In 1805 the Earth was the most certain base. By 1890, too much was known of variations in the Earth to maintain that belief, and the metal bar was much better understood and altogether more certain. In 1983, the properties of light were thought to be better known than the length of an expanding, contracting, disintegrating metal bar, and another change was made. In our modern economy, agricultural production for all its problems is relatively more stable than in the 17th century, and our beleaguered penny is infinitely finely divisible. In 1665 the penny was the constant, divisible only on a very limited discrete scale, and the natural choice of standard went the other way. In both cases the choice of standard was fundamentally statistical: the standard is that choice which is most accurately measured and most accurately transported, conveyed to whatever use it may be put.

### Statistics and the Definition of Standards

The other aspect of the relationship of statistics and standards, that I will comment on before moving to my final example, is the curious way a lack of a commonly understood standard has impeded the development of statistics in the past. I give two historical instances. Jacob Bernoulli and Thomas Bayes both published posthumously, and some historians have made excuses for them that amounted to this: “They just didn’t get around to it.” I reject that excuse. Both of them were first-rate minds who wrote their works well before they died, or so we now believe. Both of these posthumous works are now acknowledged to be masterpieces. I believe they both deferred publication for the same reason—the lack of a standard of reference that could tell them how close to certainty is “good enough.” Put crudely, they lacked the functional equivalent of a widely accepted 5 % level as a criterion—a standard of my second type, of tolerance. Bernoulli had proved a marvelous approximation theorem, but when he tried an example, he guessed that certainty to one part in a thousand was needed. He found himself concluding that over 25 000 trials would be needed to make a statement about the chance of rain, and that number was unacceptably large. Bayes tried to bound the incomplete beta integral in order to compute the posterior probability of an event, and his bounds were a whopping 0.15 apart. I conjecture that if these pioneers had had a commonly accepted standard of accuracy to serve as a benchmark, then not only would they have felt sufficiently confident about their results to see them through to publication, but the acceptance of the work would have proceeded much more rapidly. In conversation, Daniel Horvitz^2^ has made the intriguing suggestion that similar lack of reference standards is holding back surveys today.

### The Normal as a Standard

Let me close with a report on a curious example, one where a statistical object has itself become a standard, with some interesting consequences. Perhaps no statistical object is as well known today as the normal curve. It is celebrated in book titles as the “Bell Curve.” It is castigated in book reviews as the “Bell Curve.” For the past 20 years William Kruskal [9] and I have been researching this object, mostly with an eye on the evolution of its name.

The normal curve has enjoyed many names in many languages over the past 2 1/2 centuries. Normal and Gaussian are only the best known. The dozens of other names range from the commonplace (“the law of error”) to the colorful (“the gendarme’s hat”) to the exotic (“the exponic hillock”). Galton wrote of the curve that if the Greeks had known of it they would have deified it. With all this attention, it is not surprising that it has become a standard for statistical analysis. In fact, we discover that in 1838, about 50 years before it acquired the normal name, Augustus De Morgan proposed calling it the “standard distribution.” If you think about it, there is enough logic to that proposal that you wonder why it did not catch on. Both “normal” and “standard” are terms that convey two somewhat contradictory senses—they both may be read as meaning the “usual” or as meaning the “ideal,” and are frequently seen as both of these.

The normal distribution has given rise to other standards, in particular those two troubling terms whose importance Churchill did so much to emphasize, the standard error and the standard deviation. Incidentally, we are just past the centennial of the standard deviation. It was in 1893 that Karl Pearson shifted, in his manuscript notes, from referring to it as the “standard divergence” to the “standard deviation.”

I digress with an advertisement, a plug for a new “standard normal” I proposed in 1983 [10]. Laboring for years to convince students that the usual “standard normal curve,”

$$f\left(x\right)=\frac{1}{\sqrt{2\pi }}e^{-\frac{x^{2}}{2}}$$

was nothing to fear, and often failing because of its bewildering array of symbols, led me to think of a simple alternative:

$$f(x)=e^{-\pi x^{2}}$$

And why not? This is a normal curve (variance = 1/2π). It requires no normalizing constant, has no square root sign, no extraneous twos. And it has many other nice properties (for example, its quartiles are near ± 1/4) (Table 3). It did not catch on then, but I present it for your renewed consideration nonetheless, much in the spirit of an ingenious proposal I learned about from Churchill Eisenhart many years ago.

Statisticians are often approached by experimenters and asked, how large a sample should I take? This can lead to an extensive discussion: What is your problem? How large a difference is important? Are the measures correlated, etc., etc.? But Churchill had a simpler solution: ask no questions, just say “six.” Why take *n* = 6, I asked? Simple, he replied, the 0.975 % point for the Student’s *t*-distribution with *n* − 1 = 5 degrees of freedom is about equal to the square root of 6, and so these factors cancel in the 95 % confidence interval $\bar{X}\pm t_{0.975,n^{-1}}s/\sqrt{n}$ and $\bar{X}\pm s$ is an approximate 95 % confidence interval for the mean. Physicists often quote this interval (and have for years), and this way the statement will actually be correct, and as a bonus there is no need to bother with more data!

While it is perhaps not surprising that the normal curve has been adopted as a standard of data representation, I have occasionally been astonished to see just how far this adoption has gone. Let me give you some examples.

**Fig. 4:**
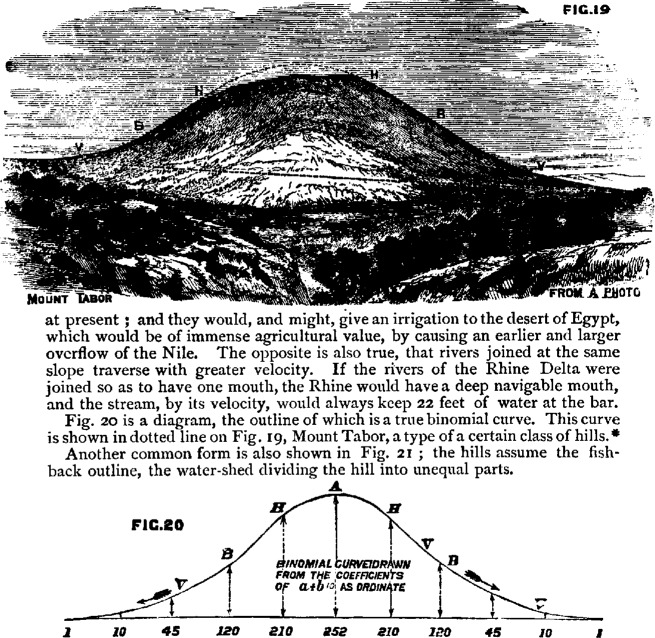
Tylor, 1875, fitting of the normal (or “binomial”) curve to a hill.

In 1875, a paper appeared with the provocative title “Action of Denuding Agencies” [11]. While the paper may not have lived up to the promise of its title (it was a paper on geological erosion), it was remarkable nonetheless. The author, Tylor, adopted as a standard the normal curve (he called it the “binomial curve or curve of denudation”), and treated deficiencies from its outline in actual hills as evidence of erosion—“denudation.” There may be an argument that could be invoked to defend this absurd procedure, but Tylor did not give it (Fig. 4).

**Fig. 5:**
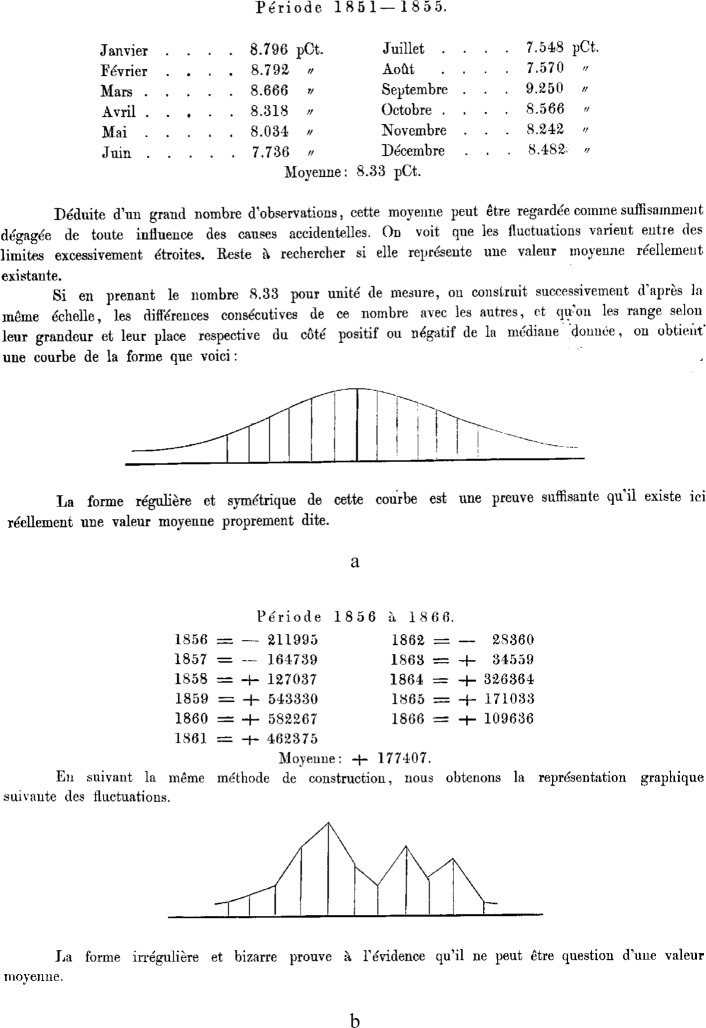
Passages from Balchen, 1869, showing two of the most egregious blunders in the history of statistics, (a) the calculation of the mean of a column of percents and its justification by a fanciful normal curve, and (b) the denial that the mean level of a times series is meaningful on the basis that the series does not follow a normal curve.

In 1869, a Swedish statistician named Balchen who was sufficiently eminent to go as his country’s representative to an International Statistical Congress at the Hague, presented a lecture on methodology that is bizarre in ways that surprise even today [12]. Balchen explained the use of the mean through a worked example. He gave Swedish data on births from the years 1851–1855, classified by month of the year, expressed as percents. Thus 8.796 % of the births occurred in January, 8.792 % in February, etc. (Fig. 5). He gave the mean percent as 8.33 %. Of course since the percents add to 100 %, the mean would have been 100 ÷ 12 = 8.33 %, even if all the births had been in January. Balchen’s comment must then be one of the greatest understatements in history: “Deduced from a large number of observations, this mean can be regarded as sufficiently free from all influence of accidental causes.” As clinching testimony for the worth of this mean, he stated that the values arrayed themselves about that mean in a normal curve. The method he used to construct that curve apparently remains a secret.

But anyone who thinks that Balchen had reached the limits of his incompetence here would be mistaken. He went on from births to trade statistics, giving the difference of exports and imports for Swedish provinces from 1856 to 1866, together with their mean. This mean, however, he judged as worthless. Why? Because when the time series was plotted, it followed a “forme irrégulière et bizarre,” not a nice symmetric normal curve!

It is comforting to see examples like this and feel that, as prone as we are now to blundering, perhaps things are improving.

### Concluding Remarks

Last May, a month before he died, Churchill wrote to me for what was to be the last time. I had sent him my obituary of W. Edwards Deming, and he was sending me his obituary of Deming for the Newsletter of the Standards Alumni Association; I expect it was the last piece he wrote. The obituary was vintage Churchill, full of facts that no one else could have known, quietly correcting errors and omissions in many other accounts of Deming, including my own. His letter closed with his characteristic sign-off—“Cherrio for now.” On this occasion I join you in saluting his memory, and with Churchill firmly in mind close with his words, “Cherrio for now…”.

## Figures and Tables

**Fig. 3a:**
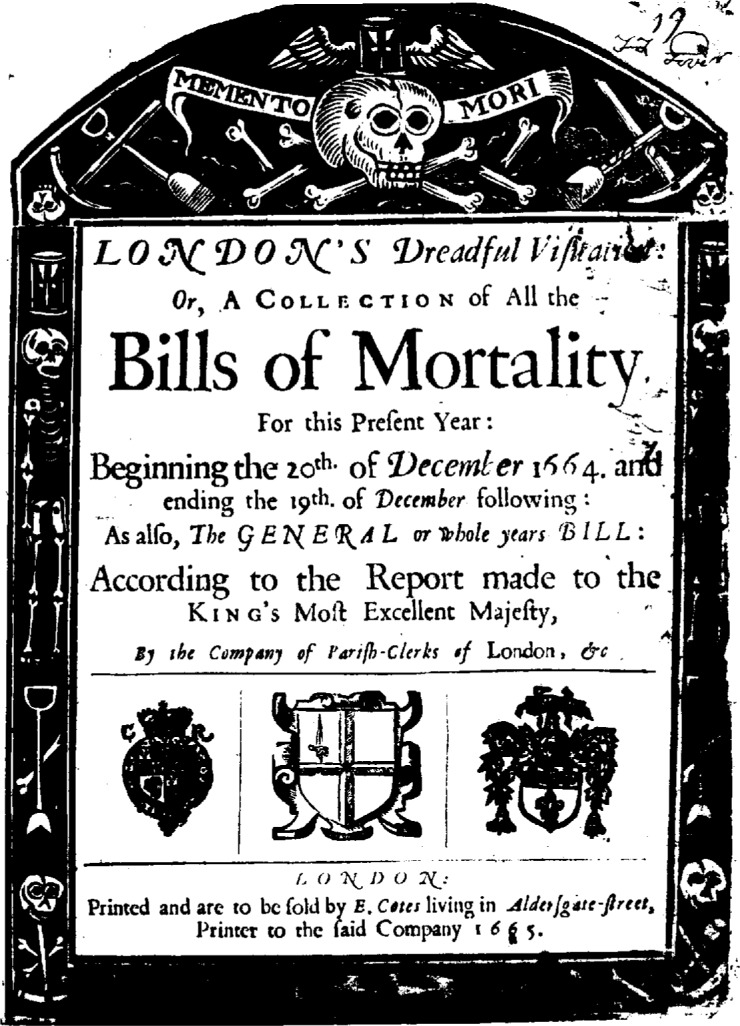
The title page of a 1665 compilation of the Bills of Mortality.

**Fig. 3b:**
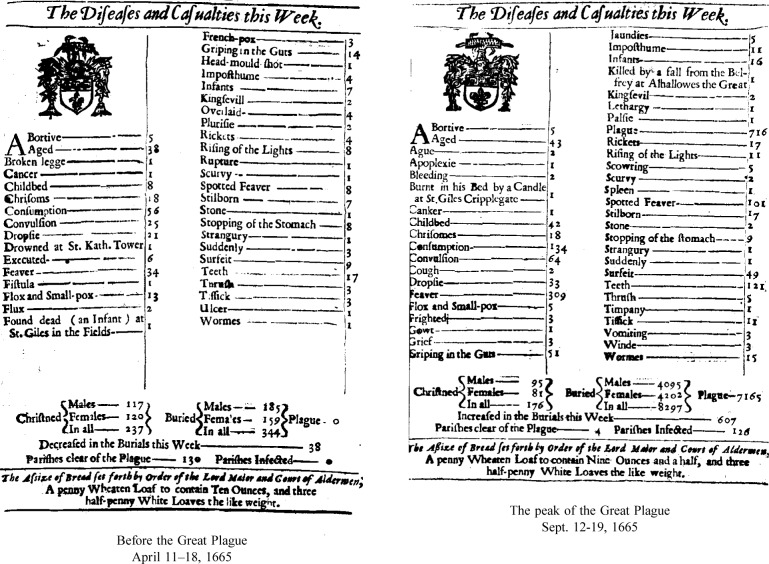
The Bills of Mortality for the weeks of April 11–18, 1665 (before London felt the force of that year Great Plague), and September 12–19, 1665 (at the peak of the Plague, when 7165 deaths were classified as due to the plague). At the bottom of each Bill there is a price index, given as the weight of a loaf of bread costing a penny.

**Table 1:** Different values reported for the Astronomical Unit (from Youden, 1972)

Number	Source of measurement and date	A.U. in millions of miles	Experimenter’s estimate of spread
1	Newcomb, 1895	93.28	93.20–93.35
2	Hinks, 1901	92.83	92.79–92.87
3	Noteboom, 1921	92.91	92.90–92.92
4	Spencer Jones, 1928	92.87	92.82–92.91
5	Spencer Jones, 1931	93.00	92.99–93.01
6	Witt, 1933	92.91	92.90–92.92
7	Adams, 1941	92.84	92.77–92.92
8	Brouwer, 1950	92.977	92.945–93.008
9	Rabe, 1950	92.9148	92.9107–92.9190
10	Millstone Hill, 1958	92.874	92.873–92.875
11	Jodrell Bank, 1959	92.876	92.871–92.882
12	S. T. L., 1960	92.9251	92.9166–92.9335
13	Jodrell Bank, 1961	92.960	92.958–92.962
14	Cal. Tech., 1961	92.956	92.955–92.957
15	Soviets, 1961	92.813	92.810–92.816

**Table 2:** Minimum height accepted for service in the military, France and Saxony (from Marx, 1906 quoting von Liebig, 1863)

Year	Height
1780	178 cm
1789	165 cm
1818	157 cm
1852	156 cm
1862	155 cm

**Table 3:** Some advantages of the new standard normal (from Stigler, 1983) [10]

Density	$f\left(x\right)=e^{-\pi x^{2}}$	$f\left(x\right)=\frac{1}{\sqrt{2\pi }}e^{-\frac{x^{2}}{2}}$
*f*(0)	1	$\frac{1}{\sqrt{2\pi }}=0.39894$
Quartile (approx.)	$\frac{1}{4}$	0.675
Mean deviation	$\frac{1}{\pi }$	$\frac{\sqrt{2}}{\pi }$
Standard deviation of the sample median	$\frac{1}{2}$	$\sqrt{\frac{\pi }{2}}$

## Footnotes

1 Member of the Statistical Engineering Division of the NIST Computing and Applied Mathematics Laboratory.

2 Daniel Horvitz is a well known statistician and past Executive Director of the American Statistical Association.
